# Purity of Drinking Water

**Published:** 1886-11

**Authors:** 


### Purity of Drinking Water.

The *Chemist and Druggist* says it is often required to give a quick indication of the freedom, or otherwise, of water from organic products. The rough and ready permanganate test cannot be relied upon. Most organic bodies, that is, those containing nitrogen, are converted into ammonia, which is ultimately oxidized into nitrous and nitric acids. The detection of nitrous acid, therefore, is important, since its presence is sufficient to condemn any water for domestic purposes. Mr. C. C. Howard has suggested a ready test, which is as follows: Into a test-glass place some of the water (not more than 50 c. c. or ℥iss.), and add a drop of hydrochloric acid, then a drop of sulphuric acid, and one of a solution of naphthylamine hydrochloride. If the water does not contain more than 1 in 100,000,000, after standing for ten minutes it should not show more than the faintest tint of pink color.

